# Key considerations for the experimental training and evaluation of cancer odour detection dogs: lessons learnt from a double-blind, controlled trial of prostate cancer detection

**DOI:** 10.1186/1471-2490-14-22

**Published:** 2014-02-27

**Authors:** Kevin R Elliker, Barbara A Sommerville, Donald M Broom, David E Neal, Sarah Armstrong, Hywel C Williams

**Affiliations:** 1 Centre for Animal Welfare and Anthrozoology, Department of Veterinary Medicine, University of Cambridge, Madingley Road, Cambridge CB3 OES, UK; 2 Department of Oncology, University of Cambridge, Box 279, Addenbrooke’s Hospital, Hills Road, Cambridge CB2 0QQ, UK; 3 The NIHR Research Design Service for the East Midlands, University of Nottingham, Nottingham NG7 2RD, UK; 4 Centre of Evidence-based Dermatology, Nottingham University Hospitals NHS Trust, Queen’s Medical Centre, University of Nottingham, Nottingham NG7 2UH, UK

**Keywords:** Prostate cancer, Cancer detection dogs, Cancer odour, Olfactory memory, Multiple sample learning

## Abstract

**Background:**

Cancer detection using sniffer dogs is a potential technology for clinical use and research. Our study sought to determine whether dogs could be trained to discriminate the odour of urine from men with prostate cancer from controls, using rigorous testing procedures and well-defined samples from a major research hospital.

**Methods:**

We attempted to train ten dogs by initially rewarding them for finding and indicating individual prostate cancer urine samples (Stage 1). If dogs were successful in Stage 1, we then attempted to train them to discriminate prostate cancer samples from controls (Stage 2). The number of samples used to train each dog varied depending on their individual progress. Overall, 50 unique prostate cancer and 67 controls were collected and used during training. Dogs that passed Stage 2 were tested for their ability to discriminate 15 (Test 1) or 16 (Tests 2 and 3) unfamiliar prostate cancer samples from 45 (Test 1) or 48 (Tests 2 and 3) unfamiliar controls under double-blind conditions.

**Results:**

Three dogs reached training Stage 2 and two of these learnt to discriminate potentially familiar prostate cancer samples from controls. However, during double-blind tests using new samples the two dogs did not indicate prostate cancer samples more frequently than expected by chance (Dog A sensitivity 0.13, specificity 0.71, Dog B sensitivity 0.25, specificity 0.75). The other dogs did not progress past Stage 1 as they did not have optimal temperaments for the sensitive odour discrimination training.

**Conclusions:**

Although two dogs appeared to have learnt to select prostate cancer samples during training, they did not generalise on a prostate cancer odour during robust double-blind tests involving new samples. Our study illustrates that these rigorous tests are vital to avoid drawing misleading conclusions about the abilities of dogs to indicate certain odours. Dogs may memorise the individual odours of large numbers of training samples rather than generalise on a common odour. The results do not exclude the possibility that dogs could be trained to detect prostate cancer. We recommend that canine olfactory memory is carefully considered in all future studies and rigorous double-blind methods used to avoid confounding effects.

## Background

Due to the high sensitivity and selectivity of the canine olfactory system and the relative ease with which dogs can be trained and handled, working dogs have been routinely used for decades as the primary means to detect a wide range of substances in environments that contain complex background odours. It has long been suspected that dogs can recognise certain aspects of human body odour. For example, bloodhounds have been used for centuries to track specific people starting from a trace of body odour on an item [1] and dogs have been trained to discriminate individual people by their scent [2,3]. However, it is only relatively recently that investigations have begun to explore the utility of detection dogs for medical diagnostic purposes, for diseases such as epilepsy [4], diabetes [5] and cancer [6-13].

The use of dogs for cancer detection is a particularly interesting technology because diagnostic methods for some cancers could be improved [14] and reliable, cost-effective, non-invasive methods of mass-screening for diseases such as prostate cancer would be valuable. It has been suggested that some forms of cancer emit detectable odours [15] and there are cases in which dogs have used olfaction to spontaneously alert their owners to what later proved to be a malignant lesion [6,7]. In one case involving breast cancer, a dog showed renewed olfactory interest some months after the tumour had been removed and a recurrent lesion was discovered in the scar tissue at the site of the operation (Sommerville and Church, personal communication). After treatment, the dog again lost interest.

Several studies have reported that it is possible to train dogs to detect or discriminate odours on the basis of cancer: bladder cancer [8], skin melanoma [9], lung cancer [10], breast cancer [10,11] and ovarian cancer [12]. However, research is at an early stage in elucidating how dogs learn these discrimination tasks and determining the best way to train them in order to produce a reliable screening capability. A review of methods and accuracy of studies to date [16] highlighted that many of these studies have yet to be fully optimised. Shortcomings have included: lack of age-matched controls potentially causing confounding of age with disease; not reporting whether an independent observer was present or a data audit completed; pseudo-replication of samples during testing, potentially making them familiar to the dogs.

The studies of canine cancer detection that have been conducted to date suggest that dogs can sometimes generalise and indicate a common cancer odour when trained with a range of samples from different donors. However, an important question is whether a potentially limited hospital supply of cancer and control samples from new, unique donors would be sufficient to train and maintain a generalised cancer detection capability.

Our study therefore aimed to test whether it is possible to train dogs to indicate an odour associated with prostate cancer in human urine, using the maximum number of training samples available from a major research hospital. We aimed to use an optimised and rigorously controlled design to rule out any possible sources of bias.

## Methods

### Animals

Ten dogs of seven different breeds (1–11 years old, four females and six males) were initially recruited from a pool of dogs attending classes at a dog training centre. This selection was based on the professional opinion of dog trainers or behavioural scientists, who had trained or observed the dogs during activities such as agility, obedience, gun-dog work, or location and retrieval of items for their owners. The dogs had not previously been used in scientific odour discrimination work. Further down-selection based on the dogs’ abilities to detect odours was carried out in the training stages (see *Training procedure*). All dogs were handled by professional dog trainers or behavioural scientists during the training and testing sessions and were cared for by their owners between sessions.

### Urine sample collection and preparation

Urine samples were collected and prepared, with the donors’ permission, in the Department of Oncology, Addenbrooke’s Hospital, UK. All samples were collected using the same protocols, at the same locations and by the same research team to ensure they had the same general background odour. The age, Prostate Specific Antigen (PSA) measurement, urinalysis, Gleason Test score (when obtained) and medical history were recorded.

In total, 50 prostate cancer (CaP) samples and 67 control samples from different individuals were collected and used over the course of the dog training period. CaP samples were collected from donors with prostate cancer that had been previously confirmed by biopsy but remained untreated. The degree of disease in CaP donors varied from small, relatively innocent tumours to metastasised cancer. Control samples were collected from men with Benign Prostatic Hyperplasia (BPH – a benign enlargement of the prostate), as well as 10 healthy men without clinical symptoms. Fifty-two controls had PSA levels <0.5 ng/ml, two had PSA <1.5 ng/ml and seven had PSA between 2.2 and 11.6 ng/ml. Thirteen of these controls, including all with PSA >2.2 ng/ml, had previously undergone prostate biopsy with negative results. Donors were excluded from the CaP group if they presented with frank haematuria or urinary tract infection, from the control group in the case of uncertain diagnosis and from both groups if they suffered from a current, non-prostatic cancer. CaP and control sample pools were chosen that were as similarly aged as was feasible based on the available urine donors.

For double-blind testing, 15 CaP and 45 control samples were used in Test 1 and 16 CaP and 48 controls in Tests 2 and 3 (Table 1). All control donors used in tests had a PSA <0.5 ng/ml. Overall mean donor ages in Test 1 were 64.1 years (standard deviation = 8.3) for CaP and 58.3 years (standard deviation = 6.6) for control. For Tests 2 and 3 the overall mean ages were 63.6 years (standard deviation = 6.4) for CaP and 57.7 years (standard deviation = 5.2) for control. The range of donor age differences between the CaP and control samples presented to the dogs in each array was: 34.5% of control samples within ± 5 years of the CaP, 28.6% within ± 10 years, 32.1% within ± 20 years and 4.8% ± 20 years or more. All samples were taken from different individuals, had not previously been presented to the dog and were presented only once during each test.

**Table 1:** Urine samples presented during tests 2 and 3

**Array number**	**Scent hole 1**		**Scent hole 2**		**Scent hole 3**		**Scent hole 4**	
1	Age	52	Age	72	Age	64	Age	56
	PSA	0.49	PSA	4.6	PSA	0.42	PSA	0.48
			Gleason	6				
2	Age	71	Age	51	Age	53	Age	59
	PSA	7.5	PSA	0.32	PSA	0.24	PSA	0.42
	Gleason	7						
3	Age	62	Age	52	Age	67	Age	57
	PSA	0.46	PSA	0.37	PSA	-	PSA	0.38
					Gleason	7		
4	Age	57	Age	58	Age	51	Age	50
	PSA	0.21	PSA	-	PSA	0.39	PSA	0.35
			Gleason	7				
5	Age	50	Age	63	Age	66	Age	53
	PSA	0.44	PSA	21.4	PSA	0.31	PSA	0.41
			Gleason	7				
6	Age	58	Age	54	Age	61	Age	59
	PSA	0.39	PSA	0.49	PSA	7.8	PSA	0.43
					Gleason	6		
7	Age	60	Age	70	Age	56	Age	58
	PSA	0.28	PSA	2.9	PSA	0.19	PSA	0.41
			Gleason	6				
8	Age	55	Age	54	Age	54	Age	67
	PSA	0.41	PSA	0.43	PSA	0.21	PSA	3.4
							Gleason	7
9	Age	68	Age	68	Age	68	Age	51
	PSA	9.6	PSA	0.43	PSA	0.40	PSA	0.15
	Gleason	7						
10	Age	54	Age	53	Age	68	Age	51
	PSA	0.47	PSA	0.21	PSA	0.16	PSA	6.3
							Gleason	7
11	Age	58	Age	69	Age	58	Age	53
	PSA	0.36	PSA	-	PSA	0.42	PSA	0.47
			Gleason	6				
12	Age	51	Age	65	Age	60	Age	60
	PSA	0.44	PSA	0.23	PSA	0.23	PSA	4.0
							Gleason	6
13	Age	61	Age	57	Age	62	Age	54
	PSA	0.40	PSA	0.49	PSA	0.32	PSA	4.2
							Gleason	7
14	Age	67	Age	64	Age	59	Age	62
	PSA	9.6	PSA	0.27	PSA	0.34	PSA	0.45
	Gleason	6						
15	Age	55	Age	65	Age	67	Age	57
	PSA	0.38	PSA	6.4	PSA	0.46	PSA	0.38
			Gleason	6				
16	Age	60	Age	63	Age	55	Age	55
	PSA	0.44	PSA	0.32	PSA	6.9	PSA	0.32
					Gleason	6		

Cancer samples are those with PSA >0.5 and Gleason score. Note the order of samples differed in Test 3.

Urine samples were collected in 50 ml polypropylene screw-cap tubes and frozen at -20°C within 10 minutes. Samples were transported to the testing centre on dry ice, defrosted in a 37°C water bath, aliquoted into 1.5 ml polypropylene micro-centrifuge tubes and stored in a freezer at -20°C. Samples were generally stored for 1 to 60 days prior to presentation to the dogs, though some were stored for up to 6 months. During training or testing, 1 ml aliquots were heated to 37°C in a water bath and then presented to the dogs in new open-top, polypropylene test tubes.

### Experimental setup

**Figure 1:**
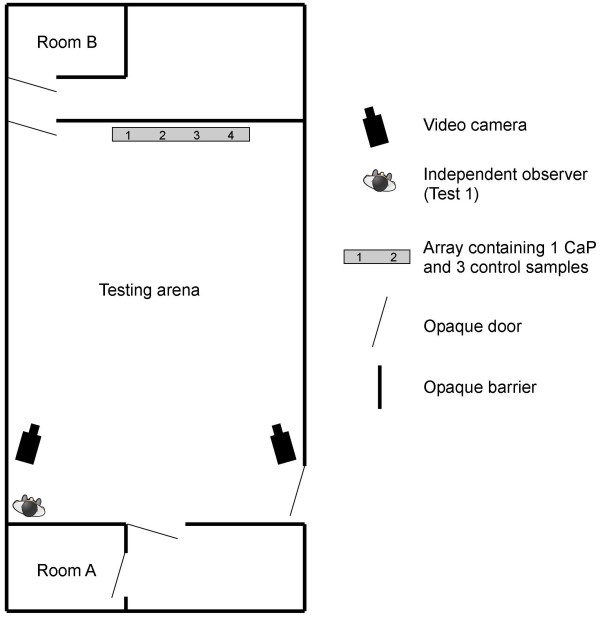
**The testing arena.** The dog and handler were visually isolated in room A while the investigator inserted urine samples into the array. The investigator then moved into room B while the handler entered the arena and allowed the dog to sniff the array.

Dogs were trained in a 6 m × 10 m rubber-floored arena (Figure 1). Urine samples were presented in four, 90 mm-deep aluminium flasks recessed into a 3 m long floor-mounted plastic array. The urine was not visible or accessible to the dogs other than by olfaction through four, 20 mm-diameter ‘scent holes’, spaced 0.75 m apart, positioned directly above each flask. These holes were labelled above with numbers. To prevent cross-contamination, the investigator wore nitrile gloves when handling sample tubes and inserted them into the array using stainless steel forceps.

### Dog training procedure

Dogs were trained using a positive reinforcement ‘clicker’ technique and food rewards/praise. Initially, food rewards were randomly hidden in one of the scent holes and the trainer rewarded the dog for sitting or lying next to and placing its nose on the hole. Once interested in the holes, Stage 1 commenced in which dogs had to indicate on single CaP urine samples placed in a random hole, with empty test tubes in the remaining holes. The criterion for a dog to move to the next stage was 9/10 successive runs correct. In Stage 2, the arrays contained one CaP sample, with the remaining holes containing different control samples. The trainer was blind to the sample positions and had to call out the number of the hole suspected to contain the CaP based on the dog’s choice. The investigator, who was visually isolated, then informed the trainer whether the choice was correct, allowing the trainer to reward the dog appropriately. When the dog was able to identify the scent hole containing the CaP more frequently than expected by chance it was moved to the formal test stage.

The number of urine samples presented to each dog during Stages 1 and 2 varied depending on their individual rate of progress. Urine samples from different donors were used to try to encourage the dogs to generalise on a common cancer odour. CaP and control samples from new donors became available in batches of 5 to 10 at intervals over the training period, and it was sometimes necessary to present urine from the same donors several times during training. Urine samples from two or three different donors were sometimes pooled in different combinations to try to vary the odour profiles. Although four scent holes were used during the testing stages, various numbers (between three and six) were trialled during initial training.

### Testing procedure

Following training, three rigorous double-blind tests were conducted for two dogs that showed ability to discriminate CaP and control samples during training Stage 2. Test 1 involved dog A, a nine year-old yellow Labrador, who had undergone approximately 5 months of training in Stages 1 and 2 prior to the test. During the test, the dog was presented with 15 arrays, each containing one CaP sample and three controls. Each of the 15 CaP and 45 control samples were from unique donors and were new to the dog. For each of the 15 arrays, the position of the CaP sample in the array (1, 2, 3 or 4) was secretly allocated using random number lists generated remotely. This sample allocation code was sent to the urine collection centre, where it was used to prepare the 15 arrays of samples. The sample allocation code was concealed from the investigators and dog handlers, who did not know the position of the CaP and controls at any point before or during the test.During Test 1, the dog handler and dog were visually isolated in room A (Figure 1), while the investigator placed samples in the array. The investigator was then visually isolated in room B and called “ready”, signalling that the handler could enter the arena and allow the dog to sniff the array. Once the dog had chosen a hole, the handler rewarded the dog and called out the position in which he/she believed the CaP sample resided (1, 2, 3 or 4). Following a run the handler and dog moved back into room A while the next array was prepared. An independent referee who did not have a vested interest in the project was present to verify the double-blinding procedure.

Tests 2 and 3 followed the same protocol except that 16 arrays of new samples were used. Test 2 again involved dog A, who had undergone approximately 8 months of Stage 2 retraining following Test 1. For Test 2, an additional independent referee was provided with the sample allocation code prior to the test. Using a live speaker-phone system this referee, who was not present at the testing centre, was able to directly respond to the position numbers called out by the dog handler and immediately inform them whether the choice was correct. Unlike Test 1, this system allowed the dog handler to reward the dog only for correct choices of the CaP samples whilst still ensuring effective double-blinding.

Test 3 involved dog B, a three-year old Border Collie with a different handler from Tests 1 and 2. This dog had undergone approximately 5 months of training in Stages 1 and 2 prior to the test. Test 3 followed the protocol for Test 2, except that it took place in a different testing venue and the visually-isolated investigator informed the handler whether the dog’s choices were correct or incorrect. Dog B was tested using 16 sets of 4 urine samples from the same urine donors who had provided the samples for Test 2, presented in a different, randomised order.

### Statistical analysis

For Tests 1 to 3, the primary outcome measure was each dog’s overall success rate in identifying cancer urine samples, compared with the success rate expected by chance, i.e. 25% (four scent holes). Each test had a power of greater than 80% to detect the difference between a dog’s success rate of 70% compared with the success rate expected by chance, assuming a two-sided significance level of 1%. The sensitivity and specificity were calculated for each test and the corresponding 95% confidence intervals were calculated using a robust variance estimate to allow for between-array variance. Agreement between dogs A and B in Tests 2 and 3, in which samples from the same urine donors were presented, was assessed using Cohen’s Kappa.

### Ethical approval

The University of Cambridge, Department of Oncology local ethics committee approved the study.

## Results

During Stage 1, four dogs were able to locate single, familiar CaP samples more frequently than expected by chance (2-tailed Binomial tests, *N* = 10, *P* < 0.05), but one of these dogs remained inconsistent. Although time was invested in training all of the dogs at this stage, three dogs progressed more quickly and it was felt that the others did not have the optimum temperaments for sensitive odour discrimination work. Four dogs were too excitable and did not sniff the holes consistently and three were insufficiently motivated to concentrate on the task. Only three of the dogs were therefore moved to Stage 2 of training.

During Stage 2, two of the dogs were able to discriminate potentially familiar CaP samples from controls with the dog handlers blind to the sample positions (2-tailed Binomial tests: Dog A, *N* = 99, *P* < 0.0001; Dog B, *N* = 48, *P* < 0.01). Dog A correctly chose the CaP sample in 75.8% of the Stage 2 runs. In the 30 runs immediately prior to Test 1 (involving CaP samples from 12 different donors), dog A correctly chose the CaP samples on 80% of occasions. Dog B correctly chose the CaP sample in 41.6% of Stage 2 runs overall. In the 30 Stage 2 runs prior to Test 1, dog B correctly chose the CaP samples on 50% of occasions. These results support the findings of other studies showing that dogs can be trained to discriminate human urine samples. However, because some of the urine samples were potentially familiar to the dogs it could not be confirmed that a cancer-related odour was being used for the discrimination.

On one occasion during training Stage 2 dog A was presented with 8 new, unfamiliar CaP and 11 new, unfamiliar control samples. Dog A correctly identified the position of the CaP sample in 6/8 runs (2-tailed Binomial tests, *N* = 8, *P* = 0.034). Although this dog seemed to be discriminating the samples on the basis of a cancer odour, a double-blind test using a larger number of new, unfamiliar samples was needed to verify the result.

During Test 1, dog A correctly indicated the position of the CaP sample for 2/15 arrays (2-tailed Binomial test: *N* = 15, *P* = 0.31), indicating that the dog was not discriminating samples based on a cancer odour. In Test 2, dog A correctly identified the position of the CaP sample in 2/16 arrays (2-tailed Binomial test: *N* = 16, *P* = 0.27). In each of these tests the sensitivity for dog A was 0.13 and specificity 0.71 (Table 2).

**Table 2:** Sensitivity and specificity of double-blind trials

**Test (dog)**	**Detected by dog**	**Prostate cancer**	**Control**	**Total**	**Sensitivity (95%CI)**	**Specificity (95% CI)**
1	Yes	2	13	15		
(Dog A)	No	13	32	45		
	Total	15	45	60	0.13 (0.03 to 0.42)	0.71 (0.65 to 0.77)
2	Yes	2	14	16		
(Dog A)	No	14	34	48		
	Total	16	48	64	0.13 (0.03 to 0.40)	0.71 (0.65 to 0.76)
3	Yes	4	12	16		
(Dog B)	No	12	36	48		
	Total	16	48	64	0.25 (0.09 to 0.52)	0.75 (0.67 to 0.82)

In Test 3, dog B correctly identified the position of the CaP sample in 4/16 arrays (2-tailed Binomial test: *N* = 16, *P* = 0.44), indicating that dog B was also not discriminating the samples based on a signature cancer odour. The sensitivity and specificity for dog B was 0.25 and 0.75 respectively (Table 2).

There was no evidence that dogs A and B were making similar choices of urine samples in Tests 2 and 3 (kappa = -0.17, 95% CI = -0.39 to 0.05), in which samples from the same urine donors were presented in different orders. There was also no apparent pattern to the choices of samples the dogs made based on the medical histories of the urine donors that provided the samples.

## Discussion

### Main findings

Previous studies have demonstrated that dogs may be able to detect odours associated with cancers, but our understanding of the best way to train and maintain dogs for this purpose is in its early stages. In this study we investigated the feasibility of training dogs to discriminate the odour of urine from men with untreated prostate cancer from controls, using as wide a range of samples as it was possible to obtain from a major research hospital that was carrying out a national prostate cancer survey. During training two dogs discriminated CaP samples from control samples more often than expected by chance, suggesting that they were recognising a signature odour associated with CaP. However, during three rigorously controlled double-blind tests involving urine samples from new donors, the dogs did not indicate CaP samples more frequently than expected by chance. Comparison of the urine sample choices made by the dogs in Tests 2 and 3 suggested that each dog was using different odour cues to select the samples.

The research team and trainers were convinced of the ability of dog A to detect a general prostate cancer odour prior to Tests 1 and 2. This result illustrates the importance of using extremely carefully controlled double-blind tests, involving the presentation of only new, entirely unfamiliar odour samples. The result does not exclude the possibility that dogs could learn to generalise based on a common prostate cancer odour if training was further optimised to achieve this.

### Confounding effects of multiple-sample learning

The most likely explanation for the discrepancy between the ability of two dogs to discriminate potentially familiar CaP samples from controls, but not unfamiliar samples, is that the dogs memorised the odour of each individual donor’s urine during training rather than generalise on a common prostate cancer odour.

We did not anticipate this because we thought that the dogs’ olfactory memory would be exceeded by the number of training samples from different urine donors. It would be useful in informing the design of future studies to verify how many odours a dog can remember and for how long. Previous studies have suggested dogs can remember at least 10 odours [17] and the minimum number of samples dog A would have needed to remember to explain its performance in Stage 2 of training was 12 CaP samples. However, the dogs may also have memorised the odours of earlier batches of training samples (both CaP and control), in which case the overall number of samples memorised would have been much higher.

Had more new samples been available for training, the dogs may eventually have exceeded the limits of their olfactory memory, encouraging them to indicate a general prostate cancer odour. In practice, we expended considerable effort on the rigorous selection of samples from a large donor study group and it would be very difficult to find a larger pool of urine donors. Another potential limitation was that it was only possible to match the ages of a proportion of control sample donors to within 5 years of CaP donors. Finding sufficient case and control samples to allow closer age matching, and also present enough samples to dogs to ensure that they generalise on a disease signature odour, may therefore always be a major limitation of studies of this type. It would be even more difficult to find sufficient donors if attempting to train dogs to detect other, rarer cancers or medical conditions.

A recent study looking at whether lateralization occurs in the canine olfactory system [18] could provide a possible solution to these issues. The study found that when sniffing novel non-aversive stimuli, dogs showed initial preferential use of the right nostril and then a shift towards use of the left nostril with repeated stimulus presentation. Assuming that human urine odours/cancer odours used in reward-based training are non-aversive to dogs, this finding may provide a quantifiable measure of whether dogs are becoming familiar with the training samples during repeated presentation (shown by a shift towards left nostril use) or whether they continue to perceive them as being novel (shown by continued use of the right nostril).

### Rewarding techniques during double-blind trials

The results of this study could also potentially have been confounded because dogs are likely to have a flexible approach to problem solving and if one strategy proves unrewarding they may try another [19]. Dog A seemed to be using a prostate cancer odour to select samples in training Stage 2, but could have changed strategy during double-blind Test 1, in which the dog was rewarded for any choice of sample, whether CaP or control. This raises the issue of how dogs should be rewarded during a double-blind test. Not rewarding the dogs at all, or rewarding them for any choice whether correct or incorrect, could result in the dogs abandoning a strategy that no longer seems fruitful and trying alternative strategies. Possible solutions are discussion in the *Summary recommendations*.

### Sample selection and presentation

Our control samples were selected on the basis of PSA level, which can be a misleading indicator [20]. There is a continuum of prostate pathology from benign hypertrophy through to a variety of types of established cancer, meaning that there could have been too much overlap between our CaP and control samples for the dogs to decipher a common prostate cancer odour. The majority of control donors had a PSA level <0.5 ng/ml, which should have ensured less than a 6% chance of them having small, clinically non-presenting prostate cancer [21]. In practice, this was the best possible control selection criteria available to us. The alternative, using controls of a much younger age who would be less likely to have prostate cancer, would have introduced the additional confounding factor of odours linked to age.

It should be noted that our sample holders did not allow the dogs to touch the urine directly and so they could only detect compounds volatile enough to be present in the headspace at 37°C. In some studies [8], the dogs could potentially make contact with the sample and hence use their vomeronasal system to detect less volatile compounds. This could also apply to the anecdotes of pet dogs alerting their owners to cancer.

### Number of dogs successful in initial training

Only three out of ten dogs initially recruited for the study passed Stage 1 of training, which limited the probability of one or more dogs successfully learning to detect prostate cancer. High failure rates such as these are common when training dogs for specialist roles because of the very specific behaviour/temperament attributes required [22,23]. It is possible that, with more time and training, more of the dogs that were initially recruited would have reached Stage 2 of training. However, within the limited time resources available, the research team focused efforts on training and testing the dogs that progressed most rapidly. It has been suggested that it may be useful to breed dogs specifically for the purpose of cancer odour detection [24] which may help to increase the proportion of suitable dogs available for future studies of this type.

### Summary recommendations

Based on our experiences, we make the following recommendations for future cancer-detection dog studies (summarized in Table 3):

**Table 3:** Summary of recommendations for future cancer detection dog studies

**Issue**	**Proposed solution**	**Evidence for effectiveness of solution**
Limited number of training samples from unique donors promotes multiple sample learning rather than odour generalisation.	Pooling of samples from different donors to create new odour profiles.	Analytical work required to validate whether pooling of biological samples is effective in creating varied odour headspace.
	Training dogs on several types of related disease odours from the outset (e.g. several forms of cancer).	Some evidence for the effectiveness of this technique based on training on two types of cancer odour [10].
	Introducing dogs to disease and control odours concurrently from the outset of training rather than introducing the disease odour in isolation first.	Some studies have successfully trained dogs by presenting cancer samples in isolation in the early stages of training. However, training on disease and control samples from the outset may reduce the risk of reliance on multiple sample learning – further validation trials required.
Methods for rewarding dogs during double-blind trials may confound earlier training.	Independent referees should provide immediate feedback to trainer on correct/incorrect responses using remote system (e.g. sample allocation code over the telephone).	Successfully employed in the present study.
	Training dogs to expect a reward only for a proportion of correct indications on positive samples.	Widely used technique in field of psychology and animal training.
Search-based discrimination tasks may not be optimal for encouraging sensitive disease odour indication behaviour.	The utility of alternative forms of discrimination task, such as habituation-dishabituation paradigms, should be explored.	Studies of novel paradigms for the measurement of olfactory discrimination in dogs and others species conducted [23].

1. Repeat presentations of samples from the same donors should be minimised as far as possible during all training. Ideally, dogs should never be presented with samples from the same donor more than once. However, in reality it may not be feasible to gain sufficient unique samples to do this for many types of cancer. Although our method of pooling of samples from different donors appeared to be unsuccessful in encouraging the dogs to generalise, it would be useful to further explore the chemical evidence for whether this could assist in creating new odour profiles to widen the pool of training samples.

2. In one study [10], dogs were successfully trained to indicate both lung and breast cancer odours. If different types of cancer have a common odour, training dogs to indicate on several different types of cancer odour in parallel may more effectively encourage generalised cancer odour detection. Training on several cancers would also facilitate amassing sufficient unique samples for training. It could also be hypothesised that some cancers may have a ‘stronger’ odour than others and that training dogs to indicate these cancers first may facilitate learning to indicate a potentially ‘weaker’ prostate cancer odour.

3. It may be that the training approach we took, in which dogs were first trained to indicate single CaP samples before having to learn to disregard control samples, biased the dogs towards learning of individual urine donors. Other studies have found that training dogs to indicate a cancer odour in isolation, before gradually introducing control samples that the dog must disregard, was successful [8]. However, we feel that if dogs are trained to disregard control samples from the outset and are never at any stage presented cancer samples without control samples being present, this is likely to reduce the risk of multiple sample learning and encourage generalisation.

4. We recommend that all future studies of cancer detection employ a system similar to that used in our tests, in which independent referees validate the double-blind testing. In order to appropriately reward dog’s choices during double-blind trials, we also recommend employing a rewarding technique similar to that used during Test 2, where the independent referee provides instant feedback via telephone as to whether the dog’s choice is correct or incorrect.

5. Over the longer-term, and especially if dogs are ever to be used for clinically screening large numbers of samples of unknown disease status, fixed/variable ratio reinforcement schedules may help to ensure the odour detection behaviour is more resistant to extinction [25]. Training dogs to expect a reward only for a proportion of correct indications on positive samples may reduce the risk of the trained cancer odour indication behaviour being abandoned in favour of a new strategy.

6. All cancer detection studies to date have employed search-based equipment that encourages dogs to choose between different samples presented simultaneously and indicate the one they believe most closely matches their training odours. Although this method may be useful for training stages, it may not be optimal for the more selective and sensitive discriminations required in a blind test situation on unknown samples. Habituation-dishabituation testing paradigms have recently been employed in canine odour discrimination research [26] and it may be that these techniques would provide a more sensitive and reliable approach.

## Conclusions

Some dogs learnt to accurately discriminate familiar human urine samples based on their odours but did not generalise to discriminate unfamiliar samples based on a signature odour related to prostate cancer. Our study illustrates that is it very easy to draw misleading conclusions about the abilities of dogs to indicate certain odours, unless extremely robust double-blind tests are conducted. Dogs may learn to memorise odours of large numbers of specific training samples rather than learn generalise based on a common odour. The olfactory memory of dogs should therefore be given careful consideration in all future studies of cancer detection ability and training and evaluation methods optimised to avoid any confounding effects due to multiple sample learning.

## Competing interests

The authors declare that they have no competing interests.

## Authors’ contributions

KRE worked with dog trainers to coordinate the experimental dog training, designed the apparatus, coordinated the double-blind testing and drafted the manuscript. BAS participated in the training and testing of dogs. SA provided sample allocation codes for double-blind testing and performed the statistical analysis. DEN coordinated the collection and analysis of urine samples. BAS, DMB, DEN and HW conceived the study. All authors participated in the experimental design and interpretation of results and inputted to, reviewed and approved the final manuscript.

## Pre-publication history

The pre-publication history for this paper can be accessed here:

http://www.biomedcentral.com/1471-2490/14/22/prepub
